# Circulating Microvesicles Are Elevated Acutely following Major Burns Injury and Associated with Clinical Severity

**DOI:** 10.1371/journal.pone.0167801

**Published:** 2016-12-09

**Authors:** Kieran P. O’Dea, John R. Porter, Nikhil Tirlapur, Umar Katbeh, Suveer Singh, Jonathan M. Handy, Masao Takata

**Affiliations:** 1 Section of Anaesthetics, Pain Medicine and Intensive Care, Department of Surgery & Cancer, Imperial College London, London, United Kingdom; 2 Intensive Care Unit, Chelsea and Westminster Hospital, London, United Kingdom; University of Technology Sydney, AUSTRALIA

## Abstract

Microvesicles are cell-derived signaling particles emerging as important mediators and biomarkers of systemic inflammation, but their production in severe burn injury patients has not been described. In this pilot investigation, we measured circulating microvesicle levels following severe burns, with severe sepsis patients as a comparator group. We hypothesized that levels of circulating vascular cell-derived microvesicles are elevated acutely following burns injury, mirroring clinical severity due to the early onset and prevalence of systemic inflammatory response syndrome (SIRS) in these patients. Blood samples were obtained from patients with moderate to severe thermal injury burns, with severe sepsis, and from healthy volunteers. Circulating microvesicles derived from total leukocytes, granulocytes, monocytes, and endothelial cells were quantified in plasma by flow cytometry. All circulating microvesicle subpopulations were elevated in burns patients on day of admission (day 0) compared to healthy volunteers (leukocyte-microvesicles: 3.5-fold, *p* = 0.005; granulocyte-microvesicles: 12.8-fold, p<0.0001; monocyte-microvesicles: 20.4-fold, p<0.0001; endothelial- microvesicles: 9.6-fold, *p* = 0.01), but decreased significantly by day 2. Microvesicle levels were increased with severe sepsis, but less consistently between patients. Leukocyte- and granulocyte-derived microvesicles on day 0 correlated with clinical assessment scores and were higher in burns ICU non-survivors compared to survivors (leukocyte MVs 4.6 fold, *p* = 0.002; granulocyte MVs 4.8 fold, *p* = 0.003). Mortality prediction analysis of area under receiver operating characteristic curve was 0.92 (*p* = 0.01) for total leukocyte microvesicles and 0.85 (*p* = 0.04) for granulocyte microvesicles. These findings demonstrate, for the first time, acute increases in circulating microvesicles following burns injury in patients and point to their potential role in propagation of sterile SIRS-related pathophysiology.

## Introduction

Severe burns injury is associated with an early onset of the systemic inflammatory response syndrome (SIRS) which is intense [1] and occasionally leads to multi-organ dysfunction. In addition to contributing to acute mortality, the development of SIRS post-burn injury has significant impacts on wound healing/repair and susceptibility to secondary infection, thereby affecting surgical outcome and long-term patient morbidity [2]. However, as with SIRS produced by infectious (sepsis) or other non-infectious/sterile causes (trauma, pancreatitis or extensive surgical insults), our mechanistic understanding of post-burn inflammation and its systemic propagation, and our ability to manipulate this response, are limited [3]. Clinical trials based on inhibiting soluble pro-inflammatory mediators in sepsis patients have failed, suggesting that although such soluble mediators function optimally in regulating local inflammatory responses, their long-range systemic activities may be fundamentally constrained by dilution, degradation and other neutralizing effects within the circulation [4, 5]. Separate mechanisms for the local to systemic propagation of inflammation may exist, and as such could provide more specific targets for the treatment of SIRS following burns injury or other etiologies that avoids unwanted suppression of local responses.

Microvesicles (MVs) are subcellular plasma membrane-enclosed particles released from activated and apoptotic cells, emerging as key indicators and long-range mediators of disease pathophysiology [6, 7]. They are released by vascular leukocytes and endothelial cells in response to a broad spectrum of microbial and endogenous stimuli. Due to their lipid encapsulation and size, MVs can function as unique biological ferries carrying a variety of molecular cargo (e.g. cytokines, lipid mediators) and conveying complex inflammatory signals to remote target cells/tissues, without the significant dilution or neutralization seen with other non-encapsulated soluble mediators. However, despite recognized roles for MVs in regulating vascular function and inflammation [8], the relationship between SIRS and acute MV release remains unclear. In sepsis, circulating levels of vascular cell-derived MVs are increased, but often only transiently [9–12], with some studies showing no change, or even decreases for some MV subtypes relative to normal levels [13, 14]. This variability may reflect dynamic changes in MV production or in MV clearance by the reticuloendothelial system and adherence to circulating cells [15]. However, it may also due to the intrinsic difficulties in studying sepsis due to its vast heterogeneous patient populations with respect to etiologies, disease onset, clinical presentation and pathophysiologies.

We hypothesized that vascular cell-derived MVs are early propagators of local to systemic inflammation in SIRS. Burn injury is a unique form of trauma with very well defined etiology, onset of insults and early clinical course, producing acute sterile SIRS in virtually all patients with a moderate to severe degree of injury [1, 3, 16]. Therefore, it may represent an optimal study population to evaluate acute MV release and its relationship to the development of SIRS. Using flow cytometry, we measured circulating levels of MVs derived from all leukocytes (CD45+), granulocytes, monocytes and endothelial cells as key vascular cell sources and representatives of the local inflammatory response following acute thermal injury. We also investigated a sepsis patient group, to compare MV levels in the single-etiology sterile SIRS group, with a more heterogeneous infectious SIRS population.

## Materials and methods

### Study population

Our study was approved by a local research ethics committee (NRES Committee London—Camden & Islington, reference 12/LO/1543). Written informed consent was obtained for all participants with capacity. In participants lacking capacity, written informed assent was obtained from a personal or professional legal representative. Retrospective consent was obtained where possible for these participants. Consent or assent was documented using a specific form, approved by the research ethics committee. All participants were over 18 years of age and were excluded if pharmacologically immunosuppressed pre-injury, or known to be infected with blood-borne viruses. All thermal injury burns patients admitted to the burns Intensive Care Unit (ICU) at Chelsea and Westminster Hospital from August 2013 to January 2015 were screened for eligibility and excluded from the study if they had injuries judged too extensive for active treatment. Patients with severe sepsis were recruited from the general ICU. As a first of its kind investigation into circulating MV levels in burns patients with effect size unknown, no pre-determined sample size criteria applied. In total, 15 burns patients, 15 sepsis patients and 12 healthy volunteers were studied.

### Clinical Evaluation

Baseline demographic data collected included age, sex, Abbreviated Burn Score Index (ABSI), Belgian Outcome in Burn Injury (BOBI), Acute Physiology and Chronic Health Evaluation II (APACHE II) score and other clinical parameters. Clinical data were collected daily including Sequential Organ Failure Assessment (SOFA) scores, blood leukocyte counts, microbiological data, and organ support requirements. ICU lengths of stay, as well as ICU and hospital mortality, were also recorded. Sepsis and burns patients that died while on the ICU were classified as non-survivors.

### Sample collection and preparation

Initial sampling of burn patients occurred within 24 hours of injury and where possible further samples were taken on day two in the burns ICU. A single blood sample was obtained from severe sepsis patients on the ICU within 48 hours of them being diagnosed with sepsis. The sepsis group included both community and hospital-acquired infections, with variable prodrome duration. Blood (18ml) was obtained from arterial cannulas or central venous catheters, after the first 10ml of blood from each line was discarded. Blood from healthy volunteers was obtained with via a 21-gauge needle and mild tourniquet, with the first 10ml discarded. Patient and volunteer blood was transferred into Vacutainers (BD Biosciences, Oxfordshire, UK) with lithium heparin as an anti-coagulant to preserve MV counts [17] and processed immediately. Considerable care was taken in the handling of blood samples throughout the procedure, to avoid any artifactual production of MVs. Plasma (platelet-rich) was obtained by centrifugation at 200 × *g* at room temperature for 10 mins, carefully aspirating the upper layer to avoid disturbing the packed cell interface. Plasma was then stored at -80°C.

### Flow cytometry analysis

The following fluorophore-conjugated monoclonal antibodies (Biolegend, London, UK) were used as pairs or alone: anti-CD45-allophycocyanin (APC) (HI30, mouse IgG1) and anti-CD14-phycoerythrin (PE) (HCD14, mouse IgG1); anti-CD66b-PE (G10F5, mouse IgM) and anti-CD11b-APC (M1/70; rat IgG2b); and anti-CD105-PE (43A3, mouse IgG1) alone. MV populations were identified as: leukocyte-derived (CD45+); monocyte-derived (CD45+CD14+); granulocyte-derived (CD66b+CD11b+); and endothelial cell-derived (CD105+).

Stored plasma samples were thawed and a 10μl volume was combined with an equal volume of antibody diluted in 0.1μm-filtered PBS and incubated in the dark for 30 minutes at 4°C. Samples were then diluted in a volume of 1ml of filtered PBS alone, or with 0.1% Triton X-100 (Sigma, Gillingham, UK) to distinguish detergent-sensitive MVs from protein aggregates, including the fluorophore-conjugated antibodies, as described previously [18]. AccuCheck counting beads (ThermoFisher, Loughborough, UK) were added to determine MV counts, with detergent-insensitive event counts subtracted from total counts. Samples were acquired CyAn ADP flow cytometer (Beckman Coulter, High Wycombe, UK) for a standardized period of 1 minute at a minimum flow rate, which in our experience produced stable acquisition rates and reproducible MV and bead counts within a set time period. Side-scatter was used as an event trigger threshold and 1.3μm average-diameter polystyrene fluorescent beads from Spherotech (Lake Forest, IL) to define upper size limit for MVs (**S1 Fig**). Data were analyzed using FlowJo software (Treestar, Ashland, OR).

For development and validation of our gating strategy, MVs were also obtained by in vitro stimulation of neutrophils isolated by density gradient separation and dextran sedimentation. Neutrophils were stimulated in suspension (1 × 10^7^/ml, RPMI-1640) with A23187 calcium ionophore (10 μM) for 20 mins at 37°C. Following centrifugation to remove cells (200 × *g*, 10 mins), MVs were added to healthy volunteer heparinized plasma that was pre-centrifuged to remove MVs (20,000 × *g*, 30 mins), and then stained as above for neutrophil MVs.

### Statistical analysis

Data were analyzed using GraphPad Prism (version 6.07). All MV data were log-transformed before analysis. A Shapiro-Wilk test was used to determine the normality of data set for the choice of either parametric or non-parametric analyses. Group comparisons were made by Student’s *t*-tests or Mann-Whitney U tests for two groups, and by one-way ANOVA with Tukey’s tests or Kruskal-Wallis with Dunn’s tests for more than two groups. Within the subject comparisons were performed by paired tests or Wilcoxon signed rank tests. For categorical data, Fisher's exact tests were used. Correlation analysis was performed using the Spearman rank test. Receiver operating characteristic (ROC) curves were constructed for the variables that were significantly different between surviving and non-surviving burns patients. Data are expressed as mean ± SD or mean (95% confidence interval, 95% CI) for parametric data, and median (interquartile range, IQR) for non-parametric data. A two-sided p value less than 0.05 was considered statistically significant.

## Results

Burns injury patient clinical characteristics are described in Table 1. A total of 15 patients were recruited to the study, all with thermal injury burns and a total body surface area (TBSA) greater than or equal to 10%. The average time from injury to initial blood sampling was 11.0 ± 4.9 hours. No patients had a suspected co-existing infection at time of injury, but they presented elevated C-reactive protein levels and white blood cells counts (>12,000/μl in 13/15 patients), indicative of a systemic inflammatory response. Hospital mortality was 33% and all deaths occurred in burns ICU following a planned withdrawal of care: a survival curve for burns patients in the first year after admission is shown in supplemental data (**S2 Fig**). The severe sepsis group characteristics are described in Table 2. There were no statistically significant differences between the burns and sepsis groups in terms of age, gender, ICU length of stay, hospital length of stay, or mortality rates. Mean age of the severe burns group (45 ± 22 years) was somewhat lower than that of the sepsis group (58 ± 16 years, *p* = 0.07), but significantly higher than the reference healthy volunteer group (30 ± 5 years, *p* < 0.0001).

**Table 1:** Burns patient demographics, assessment scores and outcome, with survivor and non-survivor comparisons.

	All (*n* = 15)	Survivors (*n* = 10)	Non-survivors (*n* = 5)	*p*
Male sex, n (%)	11	7 (70)	4 (80)	1.00
Age [years], mean ± SD	45 ± 22	34 ± 14	66 ± 20	0.003
Inhalation injury, n (%)	8 (53)	2 (20)	5 (100)	0.007
Full thickness burn, n (%)	10 (67)	5 (50)	5 (100)	0.10
TBSA %, median (IQR)	30 (18–38)	30 (18–37)	25 (15–39)	0.85
ABSI, median (IQR)	8 (6–8)	7 (6–8)	10 (7.5–11.5)	0.03
BOBI, median (IQR)	2 (1–5)	2 (1–3)	6 (4.5–6.5)	0.004
WBC [cells × 10^3^/μl], mean ± SD	19.9 ± 8.6	17.7 ± 6.9	24.5 ± 10.5	0.15
Granulocytes [cells × 10^3^/μl], mean ± SD	16.3 ± 7.9	14.3±6.3	20.2 ± 10.2	0.18
Monocytes [cells × 10^3^/μl], mean ± SD	1.4 ± 0.9	1.2 ± 0.9	2.0 ± 0.6	0.08
CRP [mg/ml], median (IQR)	14 (1.4–26)	3.6 (1.2–23.1)	17.9 (7.8–131)	0.22
ICU stay [days], median (IQR)	15 (4–53)	15 (4–32)	12 (5–79)	0.61
Hospital stay [days], median (IQR)	30 (12–72)	42 (25–78)	12 (5–79)	0.46

**Table 2:** Sepsis patient demographics, assessment score and outcome.

	All (*n* = 15)
Male sex, n (%)	9 (60)
Age [years], mean ± SD	58 ± 16
Source of sepsis, n (%):	
Pulmonary	8 (53)
Abdominal	6 (40)
Soft tissue	1 (7)
APACHE II, mean ± SD	20 ± 9
WBC [cells × 10^3^/μl], mean ± SD	14.0 ± 8.0
CRP [mg/ml], mean ± SD	247.7 ± 121.7
ICU stay [days], median (IQR)	11 (7–22)
ICU mortality, n (%)	4 (27)
Hospital stay [days], median (IQR)	34 (23–145)
Hospital mortality, n (%)	5 (33)

**Fig 1:**
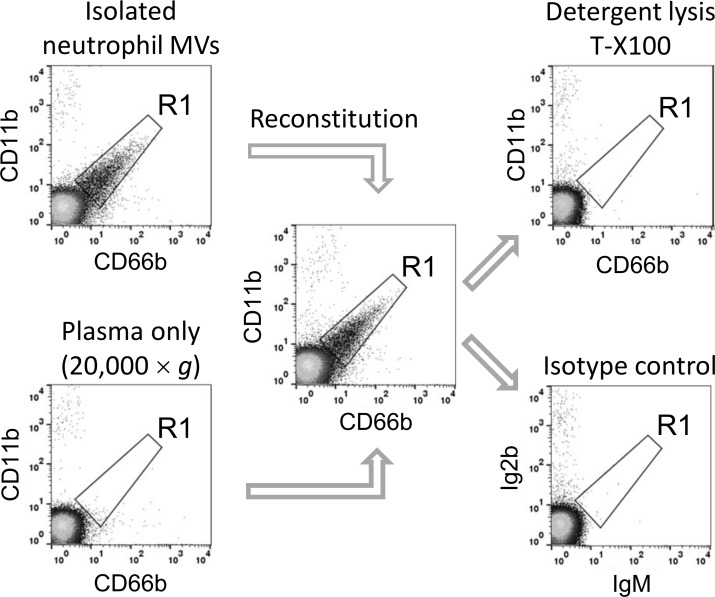
Evaluation of flow cytometry gating strategy for MVs in plasma. Suspensions of neutrophil-derived MVs and MV-depleted healthy volunteer plasma were stained with antibodies, individually or after addition of MVs to plasma (volume ratio: 1:4). Larger events were excluded using 1.3 μm beads (not shown) and a gate (**R1**) for neutrophil-derived MVs was drawn based on CD66b+, CD11b+ double-staining and a cut-off boundary defined by detergent-mediated selective lysis of MVs. Isotype-matched control staining provided confirmation of the detergent-lysis method for identification of non-MV background events.

We standardized our flow cytometry protocols for MV analysis in patient plasma using MV-free plasma reconstituted with in vitro generated MVs from isolated neutrophils. MVs were prepared from ionophore-stimulated healthy volunteer neutrophils by low speed centrifugation, and added to the donor plasma previously centrifuged at high speed to remove MVs. As shown in Fig 1, gating of neutrophil-derived MVs was based on anti-CD66b and anti-CD11b antibody staining and signal noise cut-off values defined by detergent (Triton X-100) lysis of MV, which discriminates stained MVs from detergent-insensitive particles, such as fluorescent antibody aggregates or immune complexes [18]. Isotype-matched control antibody staining confirmed the specificity and accuracy of this gating strategy. To calculate true MV counts, gated events in the detergent-treated samples were subtracted from those of the antibody-stained only sample.

Using volunteer blood samples, we also evaluated MV yields after centrifugation at the higher centrifugal force (1500 × *g*, 20 mins) that is required for the removal of platelets from plasma prior to freezing [19]. Preparation of this ‘platelet-poor plasma’ resulted in significant reduction (78.1±1.7%, paired t test, *p* = 0.0005, n = 4) in detectable neutrophil-derived MVs (**S3 Fig**). Because of this dramatic yield loss, after the initial low speed centrifuge (200 × *g*, 10 mins, to remove red and white blood cells), plasma samples were not subjected to any further centrifugation steps before freezing or during subsequent manipulations of thawed plasma. Changes in leukocyte-derived MV counts due to frozen storage were also tested using patient samples and found to be negligible (**S4 Fig**) as reported elsewhere [17, 20].

**Fig 2:**
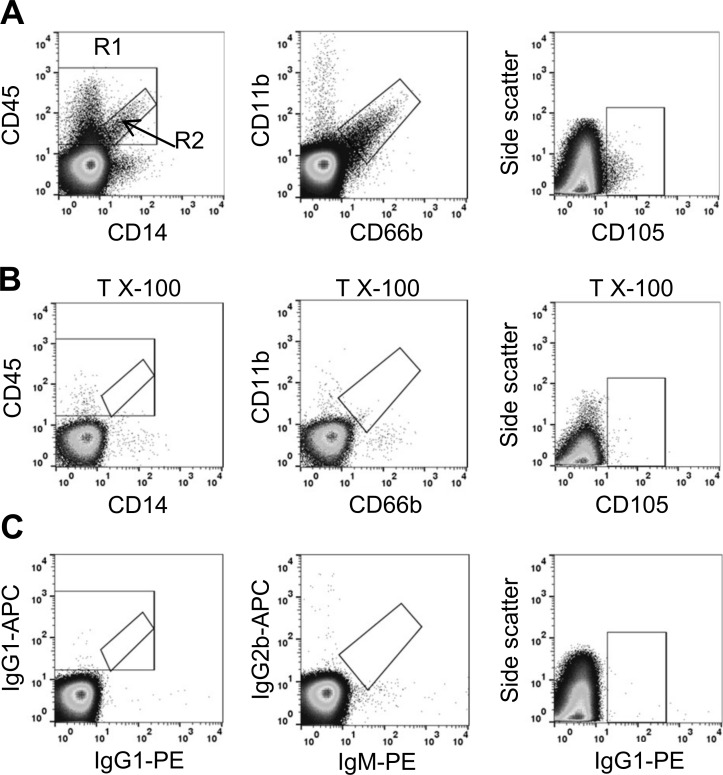
Flow cytometric identification of MV sub-types. Dots plots of burns patient plasma stained for MVs using fluorophore-conjugated monoclonal antibodies to cell-surface markers (**A**). Plots show events that have been gated on forward light scatter using 1.3 μm calibration beads to define the upper size limit. For CD45/CD14 MV analysis, total CD45+ events are shown in the larger region 1 (**R1**) and CD45+/CD14+ events in the smaller sub-region 2 (**R2**). MV identification is confirmed by sensitivity to detergent lysis (Triton X-100, 0.1%)(**B**). Matched antibody isotype and fluorophore control plots are shown for comparison (**C**).

**Fig 3:**
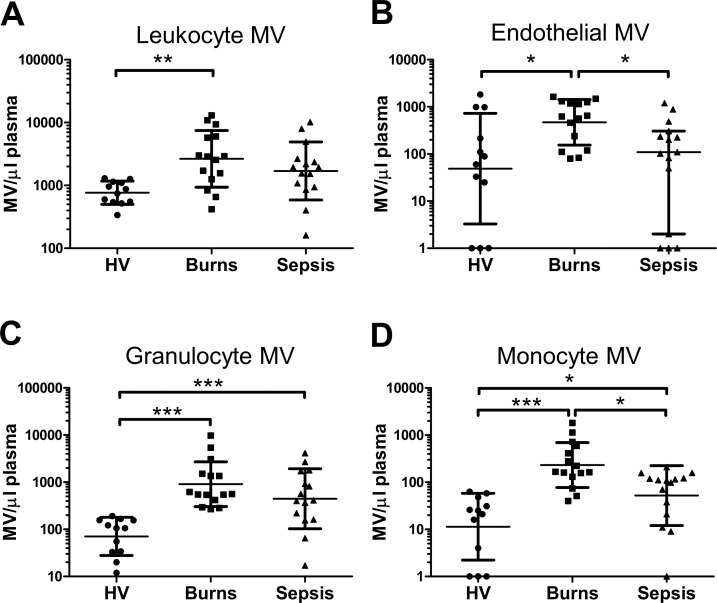
Circulating MV levels were elevated in burns patients. Plasma from healthy volunteers (HV), burns patients on day of admission, and sepsis patients was analyzed by flow cytometry to quantify MVs of different cellular origin: CD45+ leukocyte-derived (**A**), CD105+ endothelial-derived (**B**), CD66b+/CD11b+ granulocyte-derived (**C**), and CD45+/CD14+ monocyte-derived (**D**). Data are log-transformed and analyzed by one-way ANOVA with Tukey’s tests (**A**, **C** and **D;** mean ± SD) or Kruskal-Wallis with Dunn’s tests (**B;** median ± interquartile range). *p < 0.05, **p < 0.01, ***p < 0.001.

Based on these methodologies, we identified the different MV subpopulations in patient plasma samples, as illustrated in Fig 2. Burns patients displayed substantial increases (several-fold) in numbers of circulating leukocyte-derived (CD45+) MVs at admission (2,655/μl; 95% CI: 1,493 to 4,721/μl) compared to healthy volunteers (766/μl; 95% CI: 583 to 1,002/μl) (Fig 3A). Similarly, levels of detectable granulocyte MVs (CD66+/CD11b+) (907/μl; 95% CI: 495 to 1,663/μl) and monocyte MVs (CD45+, CD14+) (234/μl; 95% CI: 126 to 424/μl) were markedly increased compared to normal healthy volunteer levels (granulocyte MVs: 71/μl; 95% CI: 39 to 128/μl; monocyte MVs: 11/μl; 95% CI: 4 to 32/μl)(Fig 3A and 3D). Comparison of MV counts between arterial and venous blood samples of a subset of burns patients (n = 6) did not show any differences (**S5 Fig**). In the sepsis group, some increases were detected in all leukocyte MV subtypes compared to healthy volunteers, but the levels were overall lower than in burns patients, which in the case of monocyte-derived MVs reached statistical significance (*p* < 0.05). Endothelial-derived (CD105+) MVs (Fig 3B) were detectable in 9 out of 12 individuals in healthy volunteers, but this frequency increased to all 15 burns patients, with an overall statistically significant increase in the numbers of MVs. Endothelial-derived MVs in sepsis patients were similar to healthy controls and significantly lower than in burns patients.

**Fig 4:**
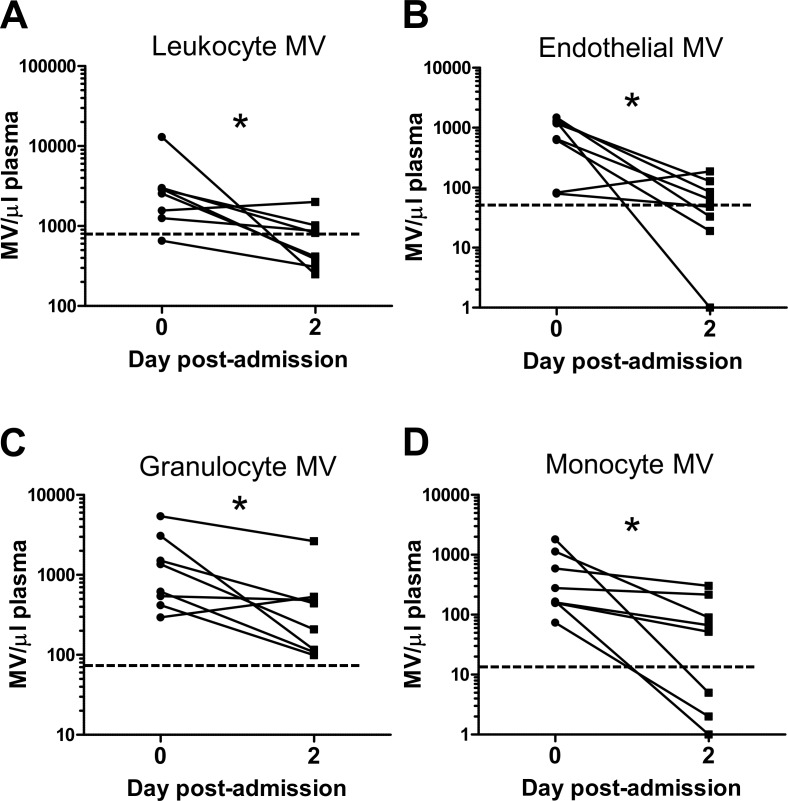
Comparison of circulating MV levels in burns patients on day 0 and day 2. Plasma from burns patients on admission day 0 and post-admission day 2 was analyzed by flow cytometry to quantify MVs of different cellular origin: CD45+ leukocyte-derived (**A**), CD105+ endothelia-derived (**B**), CD66b+/CD11b+ granulocyte-derived (**C**), and CD45+/CD14+ monocyte-derived (**D**). Data from individual patients are log-transformed and analyzed by paired t test (**A**, **C** and **D**) or Wilcoxon signed rank test (**B**). *p < 0.05.

In burns patients, no correlation was found between the levels of leukocyte-derived MVs and those of circulating parent cells (total leukocytes, granulocytes or monocytes) (Table 3), indicating that MVs are unlikely to be derived from blood leukocytes during sample collection and subsequent processing. In a subgroup of patients (*n* = 8) from which post-admission day 2 samples were obtained, there was a significant decline in all circulating MVs following the acute post-burn period (day 0) (Fig 4), indicating that their elevation may be a transient phenomenon restricted to the acute phase of injury.

**Table 3:** Spearman rank correlation coefficients for MV subpopulations in burns patients.

	Leukocyte MVs	Granulocyte MVs	Monocyte MVs	Endothelial MVs
TBSA	0.004	0.41	0.30	0.39
ABSI	0.60*	0.51	0.20	0.16
BOBI	0.61*	0.61*	0.19	-0.083
Age	0.77**	0.58*	0.36	0.057
Blood leukocytes	0.24	-	-	-
Blood granulocytes	-	0.30	-	-
Blood monocytes	-	-	0.40	-

* p < 0.05

**p < 0.001

**Fig 5:**
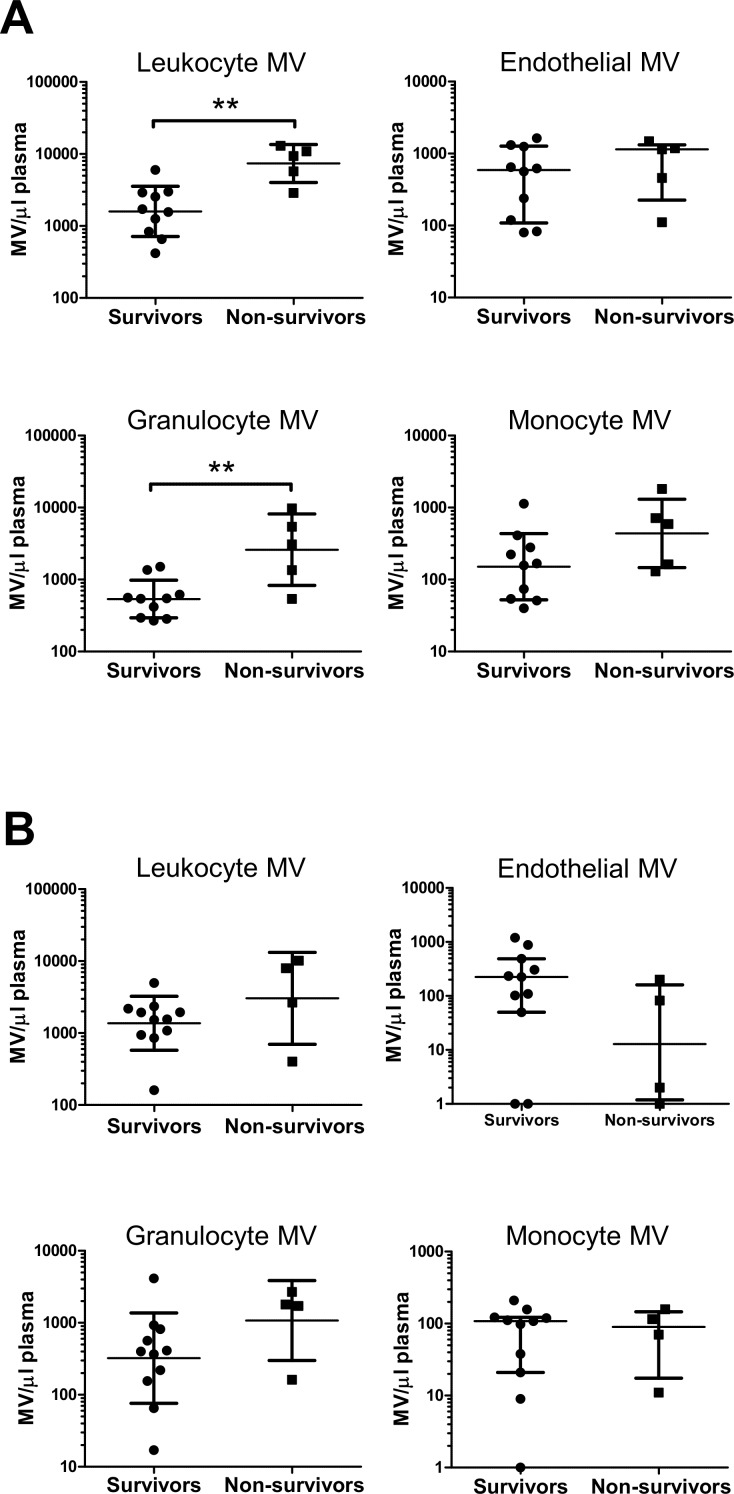
Comparison of circulating MV levels in burns and sepsis patient survivors and non-survivors. Levels of MV subtypes were compared between burns patients that recovered (survivors, n = 10) or died in burns ICU (non-survivors, n = 5) (**A**) and sepsis patients that recovered (n = 11) or died (n = 4) in the general ICU (**B**). Data are log-transformed and analyzed by t tests (for burns: leukocyte-, granulocyte- and monocyte-derived MVs; sepsis: leukocyte- and granulocyte-derived MVs; mean ± SD) or Mann-Whitney U tests (for the remainder; median ± interquartile range). Levels of total leukocyte- and granulocyte-derived MVs were higher in burns non-survivors than survivors, **p < 0.01.

Total circulating leukocyte-derived MV numbers on burns ICU admission (Fig 5A) in non-surviving patients were 4.6 (95% CI: 2.2 to 9.9)-fold higher than in surviving patients (t test, *p* = 0.002), while mean blood leukocyte cell counts were only 1.4 (95% CI: 0.6 to 2.12)-fold higher (t test, *p* = 0.15). Likewise, granulocyte-derived MVs were 4.8 (95% CI: 1.2 to 20.0)-fold higher in non-survivors (t test, *p* = 0.003), but only 1.4 (95% CI: 0.5 to 2.3)-fold higher for blood granulocyte cell counts (t test, *p* = 0.18). For monocyte-derived MVs the increase was less prominent with 2.9 (95% CI: 0.74 to 11.3)-fold changes (t test, *p* = 0.09), and for endothelial-derived MVs there was no apparent difference between survivors and non-survivors. Levels of the acute phase marker, CRP, although higher in non-survivors [17.9 (IQR 7.8–131.0) mg/ml] than survivors [3.6 (IQR 1.2–23.1) mg/ml], were not statistically different (Table 1). In sepsis patients (Fig 5B), leukocyte MV numbers (total, granulocyte or monocytes) were effectively similar between survivors and non-survivors, while endothelial-derived MVs were marginally lower in non-surviving patients.

**Fig 6:**
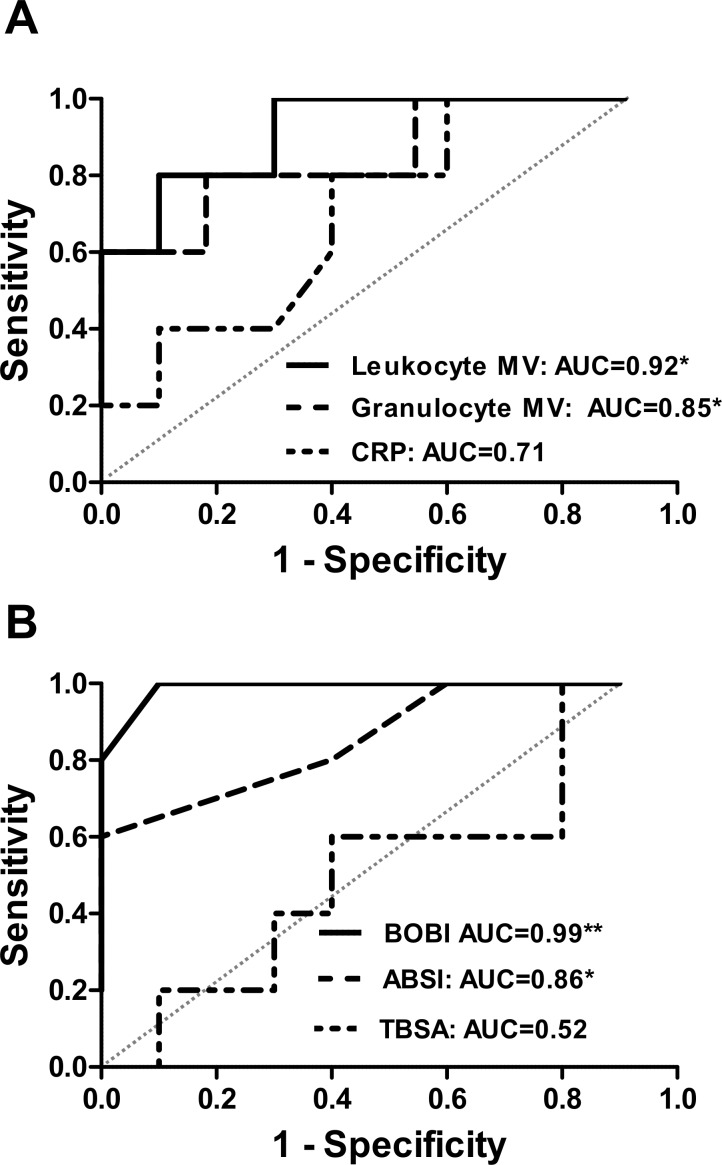
Receiver operator characteristic curves for circulating leukocyte MVs in burns patients. Receiver operating characteristic (ROC) curves and corresponding area under the curve values for: leukocyte-derived MVs, neutrophil-derived MVs and CRP (**A**), and for BOBI, ABSI and TBSA (**B**). *p < 0.05, **p < 0.01.

To assess whether MV levels are predictive of mortality in burns patients, area under the ROC curve for discriminating between survivors and non-survivors were determined, with values obtained of 0.92 (*p* = 0.01) for total leukocyte-derived MVs and 0.84 (*p* = 0.04) for granulocyte-derived MVs (Fig 6A). These values compared favorably with those for ABSI of 0.86 (*p* = 0.03) and for BOBI of 0.99 (*p* = 0.003) (Fig 6B). Moderate levels of correlation were found between leukocyte-derived MVs and ABSI (r = 0.60, *p* = 0.02) and BOBI (r = 0.061, *p* = 0.02) (Table 3). Significant correlations were also found between patient age and levels of leukocyte MVs (r = 0.77, *p* = 0.0008) and granulocyte MVs (r = 0.58, *p* = 0.02) (Table 3). In contrast, despite a similar range, age did not appear to affect elevation of circulating MV levels in sepsis patients (leukocyte MVs: r = -0.28, *p* = 0.32; granulocyte MVs: r = -013, *p* = 0.65). Burns patients with inhalation injury (survivors and non-survivors), leukocyte MV counts were 2.9 (95% CI: 1.1 to 7.1)-fold higher (t test, *p* = 0.046) and for granulocyte MVs 2.2 (95% CI: 0.6 to 8.2)-fold higher (t test, *p* = 0.18).

## Discussion

Changes in circulating MV levels and their cellular sources have previously been described in sterile and infectious SIRS patients, but until now such information has not been available for SIRS following acute thermal injury. In this traumatic injury population, which is low in incidence but induces rapid and persistent SIRS in effectively all patients with severe injury [16], we found that MVs of leukocyte and endothelial origin were uniformly elevated on the day of patient admission to the burns ICU. Moreover, increases in leukocyte-derived, and more specifically granulocyte-derived MVs, but not endothelial cell-derived MVs, were found to correlate with clinical assessment scores and be predictive of patient mortality, pointing to a role for MVs as determinants of severity in this, and potentially other, sterile SIRS conditions.

In the acute period following severe thermal injury (< 24 hours), leukocyte-derived MV levels were increased substantially (several-fold) above normal levels in healthy volunteers, but were reduced by day 2. Importantly, no correlation was found between leukocyte-derived MV numbers and their respective blood leukocyte counts, indicating that changes in circulating MVs represent an independent measure of inflammation and not merely a byproduct of SIRS-related variations in the circulating leukocyte count. Transient (<24 hours) elevations in circulating MV counts have also been described for annexin V-binding MVs in traumatic brain injury patients [11], granulocyte (CD66b+) MVs in meningococcal sepsis patients [10] and annexin V-binding and monocyte (CD14+) MVs in healthy volunteers following a bolus endotoxin injection [21]. In this study, samples from sepsis patients were obtained here over a 48 hour period post-ICU admission, which may account for the less consistent elevation of MV levels as compared to burn patients’ samples. Thus, circulating MV levels during SIRS can be very dynamic, depending on various factors including the intensity of insults and changes in balance between MV production and clearance over the clinical course, indicating the importance of timing of measurements in well-defined study populations.

In mechanical trauma patients, studies have been focused largely on platelet or ‘pro-coagulant’ (phosphatidylserine-exposed) MVs, with little information reported on other MV populations or the relationship between MVs and systemic inflammation. An inverse correlation was reported between circulating annexin V-staining (pro-coagulant phenotype), CD41+ platelet-derived MVs and patient mortality [12], while conversely and rather confusingly, injury severity has also been correlated to higher levels of total annexin V-staining MVs [22]. In the present study we did not evaluate platelet-derived MVs, because our primary focus was to investigate the roles of MVs in the acute inflammatory response, rather than coagulopathy. Hypercoagulability is an important component of severe burns pathophysiology, and indeed a recent animal study using mice has reported acute increases in circulating pro-coagulant platelet-derived MVs following a scalding injury [23]. However, coagulopathy may not be yet present at patient admission as part of the acute trauma response [24, 25] and therefore temporally distinct from acute onset SIRS. Reports of MV types other than platelet-derived or pro-coagulant MVs appear to be very limited for mechanical trauma and not present for burn patients. Ogura *et al*. showed marginally enhanced ‘endothelial-derived’ MVs in a mixed group of trauma and sepsis SIRS [26]. The markedly elevated levels of leukocyte-derived MVs we observed during early-onset sterile SIRS, indicates that further analysis in mechanical and surgical trauma patients is warranted, with study designs and analyses that take into account heterogeneity of the injury and clinical course related to onset of SIRS and organ injury.

We found that total leukocyte- and granulocyte-derived MV counts were significantly higher in non-survivors than survivors in burns patients, were predictive of mortality and correlated with clinical severity assessment scores. Burn clinical assessment scores are based on three principle risk factors: TBSA, presence of inhalation injury and increasing age [27, 28]. MV levels correlated poorly with TBSA, although all non-surviving patients had full thickness burns compared to 6 out of 10 survivors. Presence of inhalation injury and patient age were both significantly higher in non-survivors. Inhalation injury could contribute to systemic MV release via lung inflammation, with recent evidence of MV release from the pulmonary microvasculature endothelial cells in a mouse model of mechanical stretch-induced acute lung injury [29]. Alternatively, inhalation injury could merely reflect burn type and associated severity (e.g. flame as opposed to scalding injury), and overall systemic stress and inflammation, contributing to higher systemic MV production in these patients. Age was correlated with circulating leukocyte-derived MV (CD45+) and granulocyte-derived MVs in burns. Due to comorbidities such as vascular diseases, age could be a contributor to chronic elevation of MV levels [30]. However, there was no association with age in the sepsis group and the elevation of MV levels was not sustained in burns patients following the acute post-injury period. Therefore, age-related comorbidities may not be the underlying cause of high MV levels and instead age may have a direct influence on circulating MV levels following burns. Aging has been associated with increased susceptibility to pulmonary inflammation and dysfunction in patients [31] and in an in vivo animal model of burns [32], suggesting an increase in systemic and remote inflammation. Alternatively, it is plausible that aging has an effect on rates of MV clearance from the circulation due to reduced macrophage functions such as phagocytosis [33], as has been speculated for slower removal of apoptotic cells by an aging innate immune system ^37^.

The cellular and tissue sources of circulating MVs can provide important insights into disease pathogenesis [34]. We focused on leukocytes and endothelial cells as likely sources of MVs released from the inflamed burn tissue. MV production within injured or infected tissue has been described widely in vivo, with evidence of their release into the circulation, including during the perioperative period [11, 35, 36]. Following severe burns injury, substantial quantities of cellular and tissue debris are present in circulating blood [37], thus MVs produced at, or within, the burn injury site are also likely to enter the circulation. As such, circulating MVs could provide a diagnostic window into the local inflammatory reaction. Neutrophil MVs in particular are emerging as important mediators and regulators of inflammation [38]. It is therefore plausible that circulating MVs have a direct relationship with burns pathophysiology including the development of major organ inflammation and injury. Alternatively, the well-documented anti-inflammatory properties of neutrophil MVs [38], suggest they could contribute to the suppressed immune state observed post-burn with its associated risk of infection [39].

The small sample size of the present study clearly indicates a need for caution in interpretation of the results, and establishment of the precise causal relationship between circulating leukocyte MV profiles and clinical variables/outcome will require larger patient studies, as well as further mechanistic investigations. However, as a completely novel clinical observational study on MV production in severe burns injury, we presented proof-of-concept analysis to establish the levels, range and variability of circulating MV levels in this patient group. Despite the small numbers, clear and marked elevation of circulating leukocyte-derived MV numbers was observed, and correlations were found between leukocyte-derived MV levels and a number of interrelated variables, including clinical assessment scores, patient age, presence of inhalation and patient mortality. These findings were consistent with our hypothesis that MV profiles may be more uniform in burns injury due to this being a more ‘synchronized’ SIRS population, and suggest that MVs may represent a novel pathway in the early development of sterile SIRS, with the potential to offer novel mechanistic insights into its pathophysiology and recovery.

## Supporting Information

S1 Fig Defining the forward-scatter gate limit for MVs. (**A**) The upper size limit for MVs was defined using a forward-scatter (FS) gate on 1.3μm diameter fluorescent beads (SPHERO™ Flow Cytometry Nano Fluorescent Size Standard Kit). (**B**) Forward-scatter/side-scatter (SS) profiles of all CD11b/CD66b double-positive and double-negative events in a burns patient’s plasma (platelet-rich). Dashed line indicates position of 1.3μm bead gate.
(TIF)

S2 Fig Survival curve for burns patients in the first year after admission. (TIF)

S3 Fig MV yield loss following a second centrifugation step. MVs were obtained from human neutrophils by ionophore stimulation followed by centrifugation at 200 × *g* for 10mins to remove cells. Supernatants containing MVs were then mixed with an equal volume of pre-centrifuged (20,000 × *g*, 30 mins) autologous plasma and centrifuged for a further 1500 × *g* for 20mins. MV pellets were resuspended in the original volume of 50% plasma for comparison with the single-centrifuged MV sample.
(TIF)

S4 Fig Freeze-thawing of burns patients’ plasma has minimal affect on MV counts. Platelet-rich plasma obtained from burns patients (n = 5) was antibody stained and analysed directly, or after freezing (-80°C) and thawing. Wilcoxon matched-pairs signed rank test: all non-significant, p>0.05.
(TIF)

S5 Fig Comparison of MV counts in burn patient arterial *vs*. venous blood. Blood samples were obtained at the same time from an arterial cannula or central venous catheter on day 0 (n = 6). Wilcoxon matched-pairs signed rank test: all non-significant, p>0.05.
(TIF)
