# Supplementary figures and images for: Circulating Microvesicles Are Elevated Acutely following Major Burns Injury and Associated with Clinical Severity
Source: PLoS One. 2016 Dec 9;11(12):e0167801. doi: 10.1371/journal.pone.0167801 (PMC5148002; doi:10.1371/journal.pone.0167801)

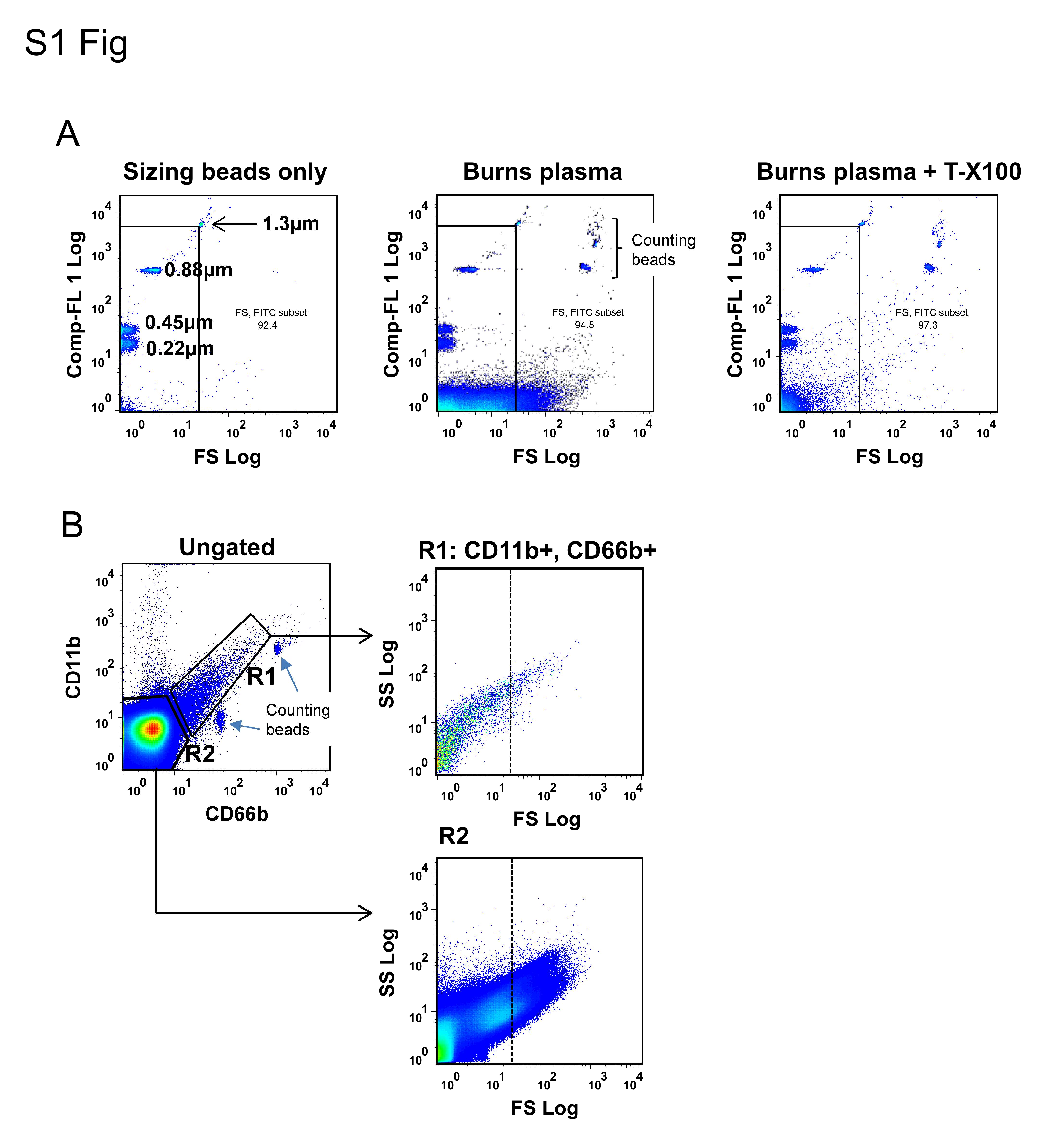

Supplement: S1 Fig — (A) The upper size limit for MVs was defined using a forward-scatter (FS) gate on 1.3μm diameter fluorescent beads (SPHERO™ Flow Cytometry Nano Fluorescent Size Standard Kit). (B) Forward-scatter/side-scatter (SS) profiles of all CD11b/CD66b double-positive and double-negative events in a burns patient’s plasma (platelet-rich). Dashed line indicates position of 1.3μm bead gate. (TIF) [file pone.0167801.s001.tif]

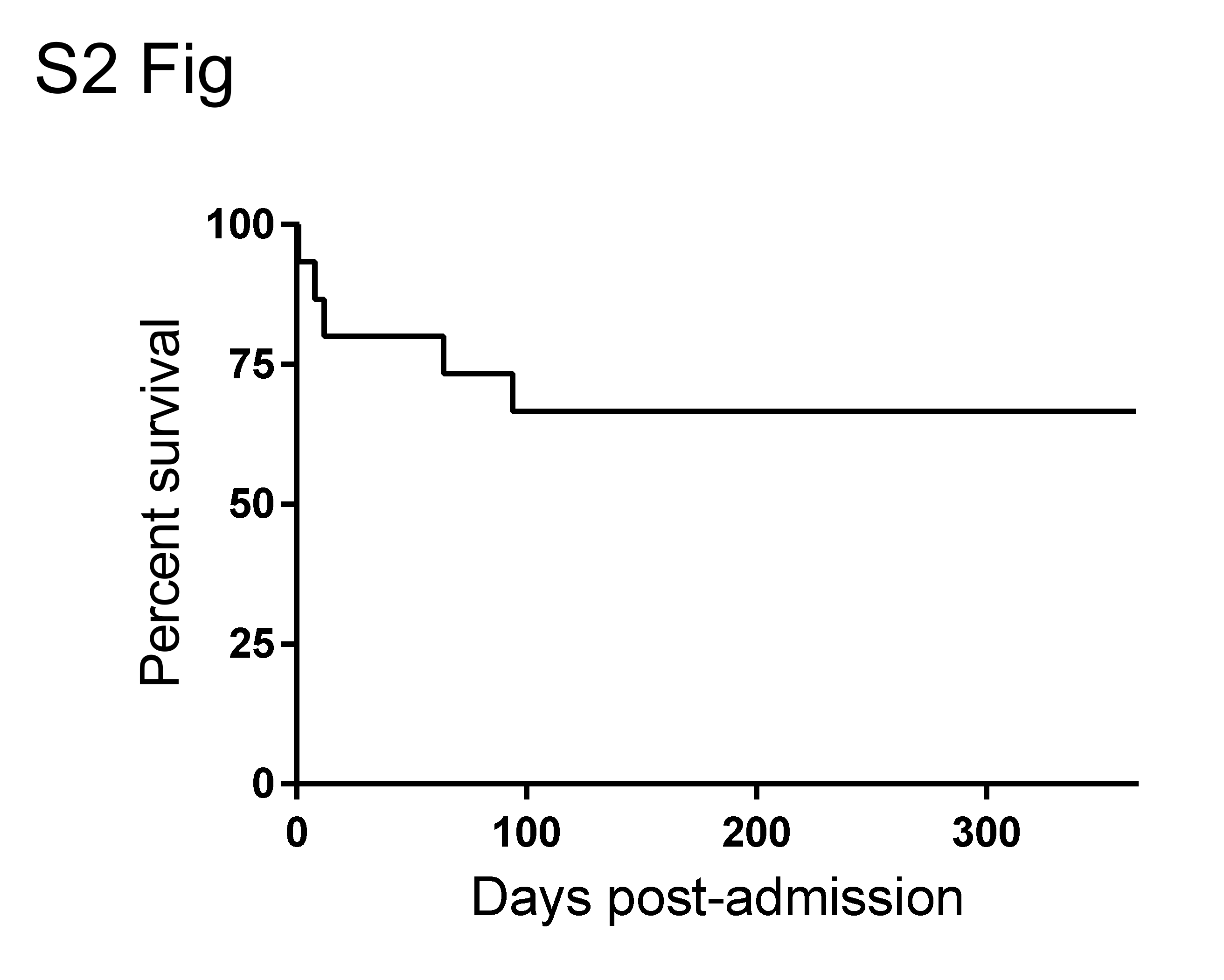

Supplement: S2 Fig — (TIF) [file pone.0167801.s002.tif]

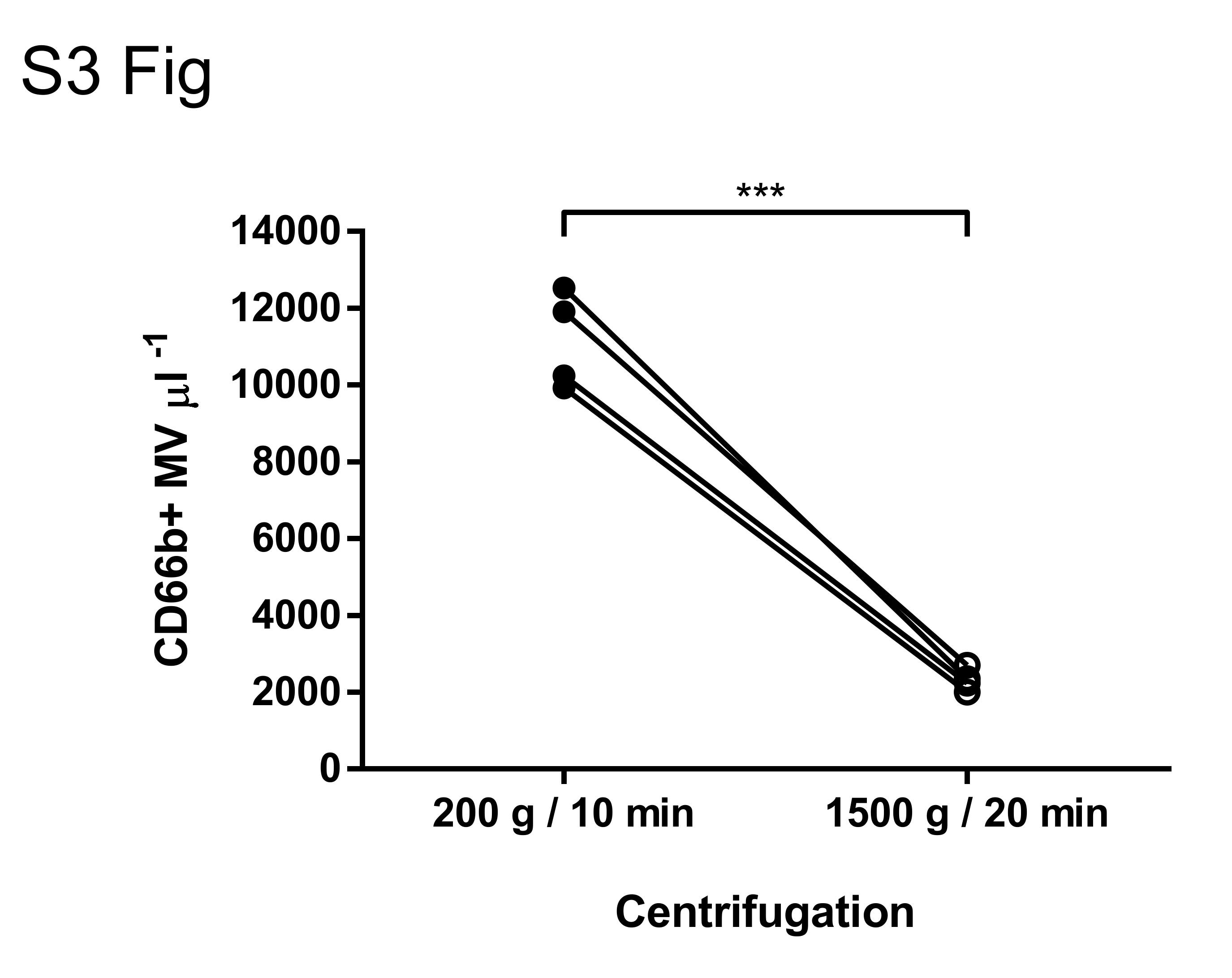

Supplement: S3 Fig — MVs were obtained from human neutrophils by ionophore stimulation followed by centrifugation at 200 × g for 10mins to remove cells. Supernatants containing MVs were then mixed with an equal volume of pre-centrifuged (20,000 × g, 30 mins) autologous plasma and centrifuged for a further 1500 × g for 20mins. MV pellets were resuspended in the original volume of 50% plasma for comparison with the single-centrifuged MV sample. (TIF) [file pone.0167801.s003.tif]

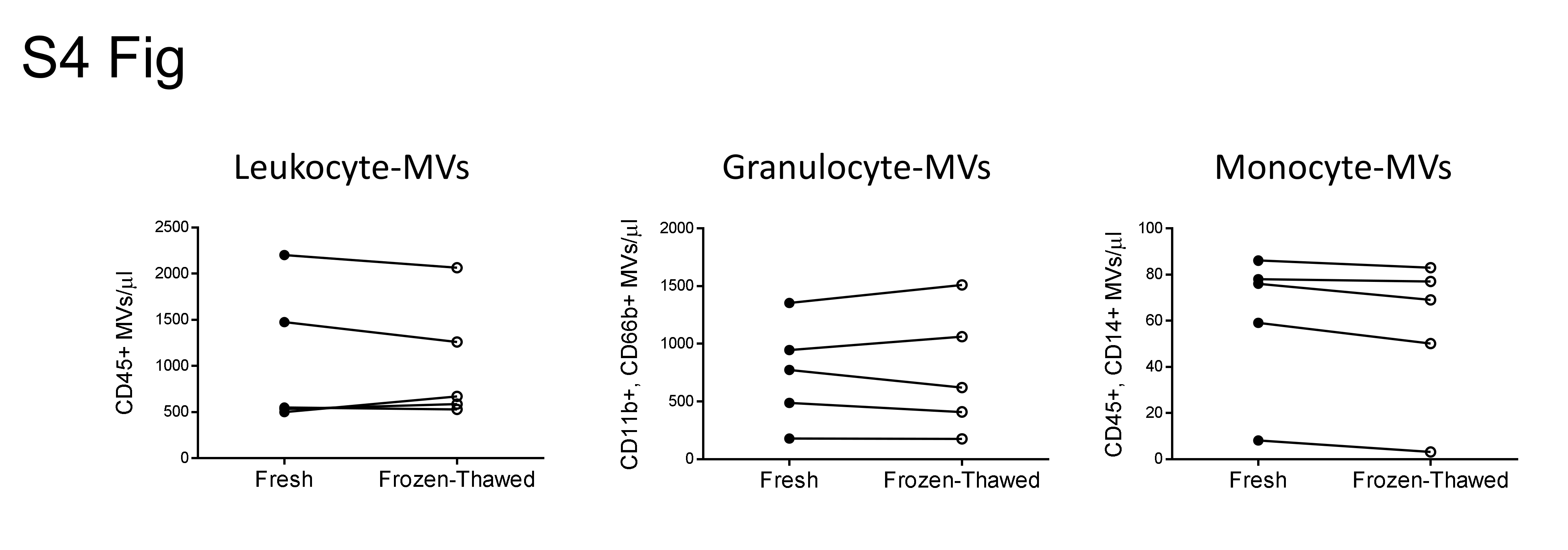

Supplement: S4 Fig — Platelet-rich plasma obtained from burns patients (n = 5) was antibody stained and analysed directly, or after freezing (-80°C) and thawing. Wilcoxon matched-pairs signed rank test: all non-significant, p>0.05. (TIF) [file pone.0167801.s004.tif]

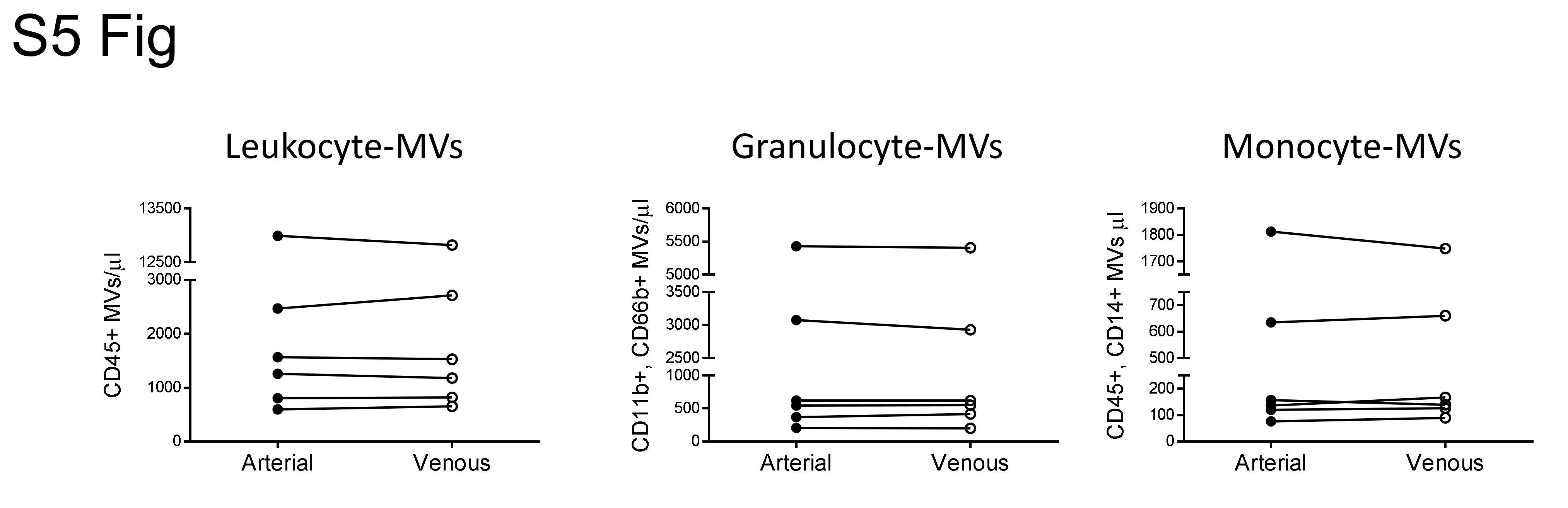

Supplement: S5 Fig — Blood samples were obtained at the same time from an arterial cannula or central venous catheter on day 0 (n = 6). Wilcoxon matched-pairs signed rank test: all non-significant, p>0.05. (TIF) [file pone.0167801.s005.tif]
